# New Approaches to Preventing, Diagnosing, and Treating Neonatal Sepsis

**DOI:** 10.1371/journal.pmed.1000213

**Published:** 2010-03-09

**Authors:** Karen Edmond, Anita Zaidi

**Affiliations:** 1 Infectious Disease Epidemiology Unit, London School of Hygiene & Tropical Medicine, London, United Kingdom; 2 Department of Pediatrics and Child Health, Aga Khan University, Karachi, Pakistan

## Abstract

Karen Edmond and Anita Zaidi highlight new approaches that could reduce the burden of neonatal sepsis worldwide.

**Five Key Papers on Preventing, Diagnosing, and Treating Neonatal Sepsis**

- Zaman K, Roy E, Arifeen SE, Rahman M, Raqib R, et al (2008) Effectiveness of maternal influenza immunization in mothers and infants. N Engl J Med 359: 1555–1564. Recent large-scale randomised trial which conclusively demonstrated that maternal immunisation can reduce serious respiratory infections in young infants and is a feasible strategy for improving health outcomes in mothers and young infants.
- Mullany LC, Darmstadt GL, Khatry SK, Katz J, LeClerq SC, et al (2006) Topical applications of chlorhexidine to the umbilical cord for prevention of omphalitis and neonatal mortality in southern Nepal: a community-based, cluster-randomised trial. Lancet 367: 910–918. The first trial to show that commonly used antiseptics could have major impacts in reducing neonatal infections and mortality in rural field-based settings in low-income countries.
- Boyer KM, Gotoff SP (1986) Prevention of early-onset neonatal group B streptococcal disease with selective intrapartum chemoprophylaxis. N Engl J Med 314: 1665–1669. The first trial to demonstrate conclusively that risk-based intrapartum antibiotic prophylaxis can prevent early-onset neonatal group B streptococcal disease in high-income settings.
- Stevens DY, Petri CR, Osborn JL, Spicar-Mihalic P, McKenzie KG, et al (2008) Enabling a microfluidic immunoassay for the developing world by integration of on-card dry reagent storage. Lab Chip 8: 2038–2045. One of a series of studies that has demonstrated that microfluidic microtechnologies can be used to create robust point-of-care diagnostic systems for low-income countries and that results can be fast, accurate, and reproducible.
- Bang AT, Bang RA, Baitule SB, Reddy MH, Deshmukh MD (1999) Effect of home-based neonatal care and management of sepsis on neonatal mortality: field trial in rural India Lancet 354: 1955–1961. The first study that demonstrated that home visiting from village health workers in the first days of life and the provision of parenteral antibiotics at home could substantially reduce neonatal sepsis and neonatal mortality in low-income countries.

Neonatal sepsis or septicaemia is a clinical syndrome characterized by systemic signs of circulatory compromise (e.g., poor peripheral perfusion, pallor, hypotonia, poor responsiveness) caused by invasion of the bloodstream by bacteria in the first month of life. In the pre-antibiotic era neonatal sepsis was usually fatal. Case fatality rates in antibiotic treated infants now range between 5% and 60% with the highest rates reported from the lowest-income countries [1]. The World Health Organization (WHO) estimates that 1 million deaths per year (10% of all under-five mortality) are due to neonatal sepsis and that 42% of these deaths occur in the first week of life [2]. There are wide disparities in neonatal care between high- and low-income countries. In high-income countries the major concern is the increasing numbers of extremely premature infants with high nosocomial infection rates due to multiresistant organisms in intensive care units. Health facility infections are also a major problem in low-income countries, but the more pressing issues are the high proportion of home deliveries in unclean environments predisposing to sepsis and ensuring that all neonates have access to effective interventions from health care providers in the first days of life^2^. Indeed, new strategies that can prevent, diagnose, and treat neonates with sepsis are needed in both low- and high-income settings.

## Pathogenesis of Neonatal Infections

Distal risk factors for neonatal sepsis include poverty and poor environmental conditions. Proximate factors include prolonged rupture of membranes, preterm labour, maternal pyrexia, unhygienic intrapartum and postnatal care, low birth weight, and prelacteal feeding of contaminated foods and fluids [3]–[5].

**Figure 1:**
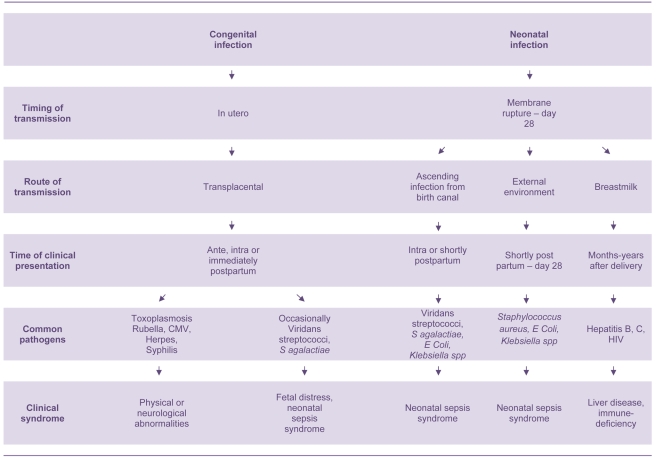
Pathogenesis of congenital and neonatal infections.

The bacteria that cause neonatal sepsis are acquired shortly before, during, and after delivery (Figure 1). They can be obtained directly from mother's blood, skin, or vaginal tract before or during delivery or from the environment during and after delivery. *Streptococcus agalactiae* (Group B streptococcus, GBS) is the most common cause of neonatal sepsis in many countries, though low rates are reported from many low-income countries, especially those in south Asia.[6]–[8]; gram-negative bacilli (*Escherichia coli*, *Klebsiella* spp., *Pseudomonas* spp., *Acinetobacter* spp.) and gram-positive cocci (such as *Staphylococcus aureus* and *Staphylococcus epidermidis*) are other important causes [6]–[8]. However, there are many difficulties in interpreting aetiological neonatal sepsis data, because many studies report selected populations of high-risk infants. Specimens from infants in the first 24 hours of life are also seriously under-represented, especially those from low birth-weight babies and babies born outside health facilities [6],[9]–[11]. Intrapartum antibiotic prophylaxis against *S. agalactiae* has also led to a substantial change in the bacteria responsible for early onset neonatal sepsis; gram-negative bacilli and *Staphylococcus* spp. predominate in countries implementing these programs [12].

There are also many other important neonatal infectious disease pathogens that are not associated with the sepsis syndrome including: *Treponema pallidum*, rubella virus, herpes simplex virus, cytomegalovirus, toxoplasmosis, *Clostridium tetani*, HIV, hepatitis B virus, and *Bordetella pertussis* (Figure 1) [1],[7],[13]. These infectious pathogens cause serious morbidities in young infants and multifaceted disease syndromes including congenital anomalies, developmental disabilities, chronic liver disease, neonatal tetanus, and apnoea. They are also important causes of morbidity and mortality in older age groups. However, only pathogens that cause neonatal sepsis are discussed in this paper.

## Neonatal Immunity

Neonates have a functionally immature immune system. They have extremely low immunoglobulin (Ig) levels except for IgG to specific maternal antigens transferred passively across the placenta during the last trimester of pregnancy [14],[15]. T cell function is relatively unimpaired but complement activity is half that of healthy adults. Neonates have a low neutrophil storage pool, and their existing neutrophils have impaired capacity to migrate from the blood to sites of infection [16].

The basal expression of Toll-like receptors (TLRs, receptors that detect the presence of microbes) is similar in the neonate and adult [17]. However, innate immune responses of neonatal mononuclear cells are characterised by markedly reduced release of the proinflammatory Th1-polarizing cytokines tumour necrosis factor-alpha (TNF-α) and interferon-gamma (IFN-γ) with relative preservation of anti-inflammatory Th2-polarizing cytokines such as interleukin 6 (IL6) [18]. These findings may reflect *in utero* requirements, including the avoidance of harmful inflammatory immune reactions [19].

These immunological problems are reflected in the clinical presentation of neonatal sepsis. Neonates have a rapid and fulminant progression of septicaemic disease, nonspecific clinical signs of infection, and difficult-to-interpret laboratory results including haematological and immunological biomarkers of infection and inflammation. Low birth-weight (preterm and small for gestational age) infants have even poorer functional immunity, and are especially at risk of sepsis [19].

However, neonates do have well-functioning cationic membrane-active antimicrobial proteins and peptides (APPs) which have microbicidal properties [15],[19]. These APPs can be found in the vernix caseosa covering the skin at birth, and in the neonatal gastrointestinal and respiratory tracts.

## Advances in Prevention

### Before Delivery

Many older studies have demonstrated that improving maternal health and nutrition before delivery is directly associated with improved neonatal health outcomes [3]. Randomised controlled trials (RCTs) of maternal protein-calorie and multiple micronutrient and supplementation have demonstrated significant improvements in rates of prematurity and birth weight and variable impact on mortality; but no studies have examined their impact on rates of neonatal sepsis [20],[21].

Maternal immunisation is an important method of providing neonates with appropriate antibodies as soon as they are born [22]. This approach is less sensitive to obstacles in accessing the health care system than are other approaches, and examples of successful interventions include maternal tetanus toxoid and influenza immunisations [23],[24]. Studies of maternal immunisation with *S. agalactiae* type III conjugate vaccine have demonstrated excellent placental transfer and persistence of protective levels in 2-month-old infants [22]. Phase I and II trials of other serotypes in nonpregnant women have also demonstrated safety and immunogenicity. A recent modelling study estimated that vaccination with *S. agalactiae* vaccine would prevent 4% of US preterm births and 60%–70% of neonatal *S. agalactiae* infections [25]. Encouraging results are also emerging from studies of maternal immunisation with pneumococcal polysaccharide and conjugate vaccines [22],[26]. The vaccines all have excellent safety profiles. However, barriers to maternal immunisation include: liability issues for vaccine manufacturers in developed countries; education of the public and health care providers regarding the benefits of maternal immunisation; and poor ascertainment of data from low-income countries [22].

### During Labour and Delivery

There is strong evidence that clean delivery practices and handwashing during delivery reduces rates of neonatal sepsis in both home and health facility settings [27]–[29]. Interventions to improve handwashing rates have been remarkably successful in research settings [30],[31]. The reasons for lack of successful scale-up of handwashing interventions into policy, programs, and behaviour change are less clear [32].

New studies from Malawi and Nepal indicate that maternal antisepsis interventions such as vaginal chlorhexidine during labour may have a significant impact on rates of neonatal mortality and sepsis in developing countries [33]. However, other studies from high-income countries have demonstrated little effect on rates of HIV or neonatal infections [34].

Intrapartum antibiotic prophylaxis has been highly effective in reducing both early-onset neonatal bacterial and maternal sepsis in developed countries [35]. Chemoprophylaxis in the US has halved the incidence of early-onset neonatal bacterial sepsis caused by *S. agalactiae* from 1.7 per 1,000 live births in 1993 to 0.6 per 1,000 in 1998 [36]. Clear protocols are in place in high-income countries for the management of women with risk factors for neonatal sepsis [37]. Risk factors for early-onset neonatal bacterial sepsis in low-income settings are probably similar to resource-rich settings, but have not been evaluated in the context of high rates of maternal undernutrition, anaemia, HIV, and malaria.

### After Delivery

There is also strong evidence that handwashing by health care providers after delivery can reduce neonatal sepsis and infection rates, especially in hospitals [27],[28]. There is less evidence for the importance of rigorous handwashing and use of antiseptics in mothers of their own infants.

In high-income settings, studies have not shown an advantage of antibiotics or antiseptics over simply keeping the umbilical cord clean [2]. However, umbilical stump chlorhexidine cleansing has recently been shown to substantially reduce neonatal deaths in Nepal [38]. Other studies investigating the effects of chlorhexidine on prevention of omphalitis are currently underway in several countries [39].

There is emerging evidence that neonatal skin antisepsis preparations such as sunflower seed oil provides cheap, safe, and effective protection against nosocomial infections in hospitalized preterm neonates and infants in studies in south Asia. Application of chlorhexidine to neonatal skin has also been shown to be effective in reducing neonatal sepsis in studies from south Asia [39],[40].

Neonatal immunisation has long been considered an important method of reducing neonatal infections. However, response varies according to the antigen [15]. BCG, polio, and hepatitis B vaccines are highly immunogenic when given at birth [41]. However, maternal antibodies interfere with a neonate's response to measles vaccine when administered under six months. Protein antigen vaccines (e.g., pertussis and tetanus toxoid) given at birth have been shown to produce poor responses compared to the same antigen given at two months of age and are associated with later tolerance [41]. Studies also indicate that *S. agalactiae* and *Streptococcus pneumoniae* vaccines are both likely to be ineffective when given in the neonatal period [15].

Breastmilk contains secretory IgA, lysozymes, white blood cells, and lactoferrin and has been shown to encourage the growth of healthy lactobacilli and reduce the growth of *E. coli* and other gram-negative pathogenic bacteria [15]. RCTs that focused on increasing early initiation and exclusive breastfeeding rates demonstrated significant reductions in diarrhoea and acute respiratory infections in neonates and older infants in India [42]. Other observational studies have demonstrated impact on infection specific mortality rates and all-cause mortality during the neonatal period [43]–[45].

Neonatal micronutrient supplementation trials have focused on vitamin A supplementation. Older studies have shown significant reductions in respiratory disease in low birth-weight infants after the administration of parenteral vitamin A [46]. More recently, trials of newborn vitamin A supplementation have shown encouraging reductions in neonatal mortality, and more trials are underway [47].

In high-income countries, clinical trials of immune stimulants such as granulocyte/monocyte colony stimulating factor (GM-CSF) to enhance the quantity and quality of neonatal neutrophils and monocytes appear promising but have not yet shown a significant clinical benefit [15]. The evaluation of recombinant APPs as adjunctive therapy for neonatal infection are still under evaluation. The impact of TLR agonists to improve defences against microorganisms are also being evaluated [15].

## Advances in Diagnosis

Neonatal clinical sepsis syndrome identification is difficult as the clinical signs of neonatal septicaemia can be very similar to those of other life-threatening diseases such as necrotising enterocolitis, hyaline membrane disease, and perinatal asphyxia [48],[49]. However, recent studies in middle- and low-income countries have provided seven danger signs which can be used to identify infants with very severe disease including neonatal sepsis (Table 1) [49]. These signs provide high sensitivity and moderate specificity for detecting serious illness in newborns in low-resource settings and have now been incorporated into the new neonatal WHO Integrated Management of Childhood Illness (n-IMCI) guidelines.

**Table 1:** Clinical symptoms and signs of severe neonatal illness including sepsis.

History of difficulty feeding
History of convulsions
Movement only when stimulated
Respiratory rate ≥60 breaths per minute
Severe chest indrawing
Axillary temperature ≥37.5°C
Axillary temperature <35.5°C

From [49].

Identification of neonatal sepsis before delivery also remains challenging. A combination of maternal risk factors and clinical signs and symptoms is currently used [50]. However, peripartum proteomic analysis of the amniotic fluid is now offering the opportunity for early and accurate diagnosis of early-onset neonatal sepsis in the select population of women undergoing amniocentesis in high-risk pregnancies [51],[52].

Confirmation of pathogenic organisms allows targeted antibiotic therapy. However, identification of pathogenic organisms in neonates with sepsis syndrome is fraught with difficulties. Bacterial load may be low due to mothers receiving antepartum or intrapartum antibiotics and because only small amounts of blood can often be taken from newborns [53]. Contamination rates may also be very high due to the technical difficulties of sterile venipuncture in small babies. There may also be misinterpretation of the role of coagulase-negative staphylococci (e.g., *S. epidermidis*), as these organisms are both normal skin flora and pathogenic organisms in preterms and infants with indwelling blood vessel catheters [54].

Automated blood culture systems have long been considered the gold standard for microbiological diagnosis. However, despite improvements in growth media and instrumentation, results of blood culture can be delayed by up to 48 hours [53],[55]. The condition of a neonate with true sepsis can deteriorate quickly, thus the most common approach is to initiate empiric broad-spectrum antibiotic therapy in all young infants with suspected bacterial infection [49]. A negative blood culture after 48 hours may allow cessation of antibiotic therapy in a well infant. While appropriately cautious, this practice leads to antibiotic exposure in a large number of newborns for whom antibiotic treatment may be unnecessary since blood cultures are positive in only 5%–10% of suspected sepsis cases, even at highly resourced facilities [56].

Antigen detection techniques allow rapid detection and identification of microorganisms without culturing. The most commonly used commercially available test is the latex agglutination assay, which is based on specific agglutination by bacterial cell wall antigens of antibody-coated latex particles. However, these tests can only detect specific organisms such as *S. agalactiae* and are associated with high false positive and negative rates [57]. New urinary antigen tests for pneumococcus are more encouraging but are also associated with false positives from pneumococcal carriage [58].

The polymerase chain reaction (PCR) has been widely used in biomedical research laboratories for pathogen identification in neonatal sepsis and in some clinical hospital laboratories. The high sensitivity of PCR allows detection of bacterial DNA even when concentrations are low [57]. Conventional assays are being replaced by a newer “real-time” system, which is faster and associated with lower contamination rates because amplification and detection occur simultaneously in a closed system [59]. The real-time PCR is based on the measurement of a fluorescent signal generated during each amplification cycle. It produces quantitative results within 30 minutes and calculates bacterial load. Broad-range real-time PCR uses a single primer to detect the universal bacterial genome (16S RNA or 23S RNA) which is a conserved ribosomal genome sequence across all bacterial genera [60]. Broad-range real-time PCR can be used to distinguish bacterial septicaemic disease from other causes of neonatal illness such as asphyxia or complications of prematurity. However, it has been used with varying success in the analysis of whole blood for neonatal sepsis; specificity is generally high but sensitivity can be as low as 40% [60],[61]. In contrast, multiplex PCR involves the parallel amplification of different targets but is focused only on specific pathogens, and false negatives can occur if the aetiologic agent of interest is not included in the database [62]. Real-time PCR is now often used to screen for microbial load, followed by sequence-based targeting and identification of PCR amplicons (pyrosequencing) [62]. This process can detect very small copy numbers of specific nucleic acid sequences. There is also a new commercially available multiplex pyrosequencing PCR assay which can identify up to 40 different bacterial and fungal pathogens directly from whole blood [63]. Real-time PCR and pyrosequencing of the universal 23S rRNA gene has also recently been used successfully in neonatal blood culture samples [64]. Further tests on neonatal whole blood have been planned by a number of different research groups.

The biggest problem with real time PCR testing is that the specimen must be collected with a sterile venipuncture, which may be difficult in young neonates. Neonatal capillary heel prick specimens are easier to collect but highly contaminated by skin flora. There is also high potential for contamination of enrichment media, reagents, or the sample during collection and processing [61] Other problems include low sensitivity due to competition from human DNA in whole blood, especially if white cell counts are high. Also, bacterial organisms require lysis before their DNA can be available for analysis, and gram-positive organisms are difficult to lyse because of their resilient cell wall [61]. Real-time PCR technologies are also expensive and currently can be used only by highly trained staff.

Important haematological tests include microscopic examination of the blood for white cells (total leucocyte count, differential, neutrophil count, and immature neutrophil to total neutrophil ratio). Advantages are that these specimens do not require sterility and a heel prick specimen can be used. However many of these indices are falsely low in a septic neonate.

Biological biomarkers are human blood components that increase in response to infection. The most commonly used acute phase reactant is the C-reactive protein (CRP). However, the CRP takes 12–24 hours to increase to measurable levels; its half life is very long and it takes 5–7 days to normalize after eradication of the infectious agent. Cytokines such as IL6, IL8, TNF-α, and procalcitonin have also been extensively studied [65],[66]. Cytokines rise quickly after infection even in neonates, and are more sensitive to low concentrations of pathogens than CRP [66]. However, cord and postnatal blood cytokine concentrations can be depressed in the presence of pregnancy-induced hypertension and can rise after induced vaginal or urgent cesarean delivery, delivery room intubation, muscular damage, and inflammation from other causes [57]. Simultaneous measurement of multiple biomarkers may improve both sensitivity and specificity [66],[67]. However, biomarker assays are likely to be less acceptable to physicians who often place higher value on tests that confirm biological agents and allow targeting of antibiotic therapy [57].

Microtechnologies, especially microfluidics, have provided the greatest recent contribution to the diagnosis of neonatal sepsis. Microfluidics is the study of the behaviour, precise control, and manipulation of fluids geometrically constrained to submillimetre (nanolitre or picolitre) channels [68]. Microfluidic technology uses the unique proprieties of continuous flow micro-volume channels: viscosity, surface tension, energy dissipation, and fluidic resistance, and also includes micro pneumatic pump and valve systems. One specific application of microfluidics is bacterial DNA protein microarray hybridization [69]. In this test, DNA probes specific to selected targets are spotted on a glass or silicon slide in a known order. Target DNA fragments are labelled with a reporter molecule, combined into a single hybrid, and measured using fluorescent signals [62],[68]. This technique has been used in the identification of the specific sepsis pathogen in bacterial meningitis, acute viral respiratory tract infections, and neonatal sepsis, and also in the detection of their antimicrobial resistance and virulence genes in research settings [63].

**Figure 2:**
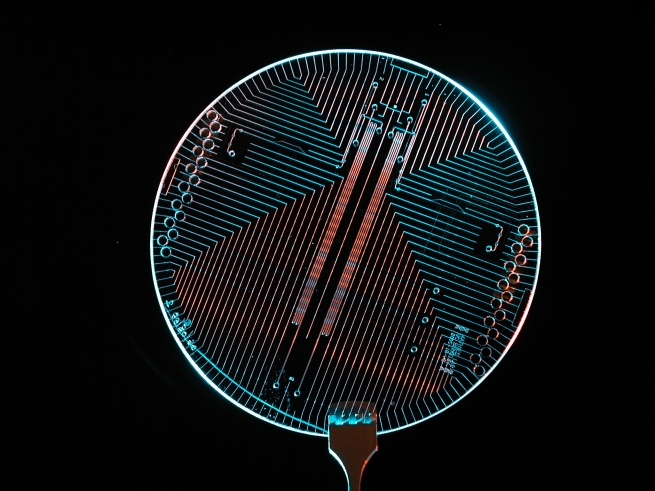
Example of “lab on a chip” point-of-care device.

Microfluidic technology has also allowed sample preparation and a number of different assays to be combined in small, disposable, single-use diagnostic cartridges or cards that have been called a “lab on-a-chip” or LOC (Figure 2) [68]. Some LOCs have combined sample preparation, biomarkers, real-time PCR, and DNA microarrays to provide information about indices of inflammation, pathogen identification, and antimicrobial susceptibility patterns at the point of care [68],[70]. LOCs have been reported to perform assays at sensitivity, specificity, and reproducibility levels similar to those of central laboratory analysers, but yet require little user input other than the insertion of the sample. Single drops of blood, faeces, and saliva have all been tested with encouraging results. LOCs are currently being evaluated for use in sepsis, endocarditis, HIV, tuberculosis, severe acute respiratory syndrome (SARS), and pneumonia [68]. However, they are not yet in clinical use nor licensed by regulatory authorities.

## Advances in Treatment

As neonatal sepsis can be rapidly fatal if left untreated, highly effective antibiotic therapy must be used and delays in the provision of care must be minimised. Treatment must be effective against the causative pathogen, safe for the newborn, and feasible to deliver reliably in the hospital or community setting.

Parenteral (intravenous or intramuscular) regimens for neonatal sepsis currently recommended by national paediatric associations are a combination of penicillin/ampicillin and gentamicin, or third-generation cephalosporins (e.g., ceftriaxone or cefotaxime) for 10–14 days. These antibiotics are safe and retain efficacy when administered at extended intervals (e.g., twice daily or daily dosing) [56]. These regimens are very effective against *Streptococcus* spp., but *Staphylococcus* spp. can be highly resistant [71]. Gram-negative antimicrobial susceptibility to ampicillin and gentamicin can also be poor, especially for *Klebsiella* spp. [8],[71]. Emerging *E. coli* resistance to ampicillin, gentamicin, and third-generation cephalosporins in hospital nurseries in both developed and developing countries is also causing increasing concern [8]. The potential for significant life-threatening toxicity among neonates associated with chloramphenicol makes it the least preferred empiric parenteral therapy [56].

Oral antibiotic therapy must be considered in settings where referral is not possible and there are no health care providers trained to give parenteral antibiotics [72]. The incremental benefit of injectable over oral antibiotics is not known, and oral antibiotic therapy is better than no antibiotic therapy at all. A series of trials are currently evaluating the impact of home and clinic-based short course (7 days) intramuscular and oral antibiotic therapy for neonatal sepsis in low-income countries [72]. Most data are available on the effect of oral cotrimoxazole in community-based treatment of serious neonatal bacterial infections from Nepal and India. However, there are concerns about high resistance rates, and side effects such as neonatal jaundice have been reported [71]. Oral amoxicillin is highly efficacious against *Streptococcus* spp. and some gram-negative bacilli and has an excellent safety record. However, it has no anti-*Staphylococcus* coverage and resistance is emerging in gram-negative bacilli such as *E. coli*. New, better-absorbed oral antibiotics are also being considered. The new second-generation cephalosporins (e.g., cefadroxil and cefuroxime) have an excellent safety profile, a spectrum of activity similar to cotrimoxazole, and may be more effective given the high resistance of neonatal pathogens to cotrimoxazole. Ciprofloxacin also is increasingly accepted as safe in neonates and warrants further investigation for treatment of infections in newborns. However, the current cost of these agents and potential for exacerbating antimicrobial resistance may limit widespread use in developing countries [72].

Poor maternal-neonatal health systems, low levels of care-seeking, and lack of access to sick newborns during the first day of life, when mortality risks are highest, are also important concerns [73]. Recent studies have shown that community health workers can deliver antibiotic treatment to neonates with very severe infections at home safely and acceptably when hospitalization is not feasible [74]. Trials are currently evaluating the effectiveness, quality of care, and coverage of these community health worker programmes in Asia and Africa [73]. Barriers to large-scale implementation include high cost, poor staff training and retention, and difficulties with referral (e.g., lack of ambulances and poor institutional links).

## Summary and Next Steps

Newborn sepsis is a major cause of child mortality across the world. Industrialized countries have made remarkable progress in reducing newborn sepsis and sepsis-related mortality by providing access to hygienic skilled delivery for all women, risk-based intrapartum antibiotic prophylaxis, and high-quality intensive care for newborns that need it. Although resource constraints preclude whole-scale adoption of these strategies in developing countries, there are a number of low-cost proven interventions and promising approaches that have the potential to significantly reduce the burden of neonatal sepsis worldwide (Table 2).

**Table 2:** Effective current measures and new approaches to prevent, diagnose, and treat neonatal sepsis.

Category	Measure	Item
**Prevention**	*Current measures*	Improved maternal health and nutrition
		Clean delivery practices and handwashing
		Risk-based intrapartum antibiotic prophylaxis
		Hand washing from health care providers
		Promotion of early initiation of exclusive breastfeeding
	*New approaches*	Maternal *S. agalactiae* and *S. pneumoniae* immunisation
		Maternal vaginal chlorhexidine other antisepsis preparations
		Neonatal protective and antisepsis skin preparations
		Neonatal vitamin A supplementation
		Recombinant active antimicrobial proteins
		Toll like receptor agonists
**Diagnosis**	*Current measures*	Blood culture
		Antigen detection
		Blood neutrophil count and differential
		C-reactive protein
	*New approaches*	Proteomic amniotic fluid analysis
		Improved clinical syndrome identification
		Real-time polymerase chain reaction
		Interleukin inflammatory indices
		Microfluidic microtechnologies
		“Lab on a chip” point of care devices
**Treatment**	*Current measures*	Parenteral antibiotics (penicillin/amoxycillin and gentamicin or third generation cephalosporins) for 10–14 d
	*New approaches*	Shorter courses of antibiotic therapy
		Better-absorbed oral antibiotics especially second generation cephalosporins and ciprofloxacin
		Programs to increase the access of neonates in remote areas to health care providers in the first days of life

However, practicability of implementing these new advances must be considered. Skilled attendance at delivery is increasing in low- and middle-income countries. Thus, intrapartum approaches such as risk-based antibiotic prophylaxis and improved hand washing during delivery are likely to be both cost-effective and feasible in these countries. More challenges face the implementation of diagnostic technologies. It may take many years for technologies such as the “lab-on-a-chip” to be sufficiently robust and affordable for scale-up to low-income countries. Home-based antibiotic treatment of neonatal sepsis also faces major obstacles to large-scale implementation. Concerns such as “one law for the rich and another for the poor” have already been raised. A careful assessment of the risks and benefits of new technologies and interventions is clearly needed. In low-income settings there are also difficulties with care-seeking for neonatal illnesses, and home visiting programs are needed to identify sick newborns early in life. Neonatal sepsis is also one of the most rapidly fulminating clinical diseases, and many practitioners, including experienced neonatologists, administer parenteral antibiotics rather than wait for the results of any diagnostic tests. These practitioners rightly consider that the individual patient's health is more important than the potential risks of emerging antibiotic resistance.

Thus, front-line health workers and families must be partners in all research and evaluation planning. Detailed assessment of end-user attitudes and preferences using formative and qualitative research methods must be included in the development of programs to reduce morbidity and mortality from neonatal sepsis. Finally, advocacy for equitable resource allocation across and within countries must be a priority and modelling techniques to assess public health impact of neonatal sepsis interventions must be developed and used more widely.

## Abbreviations

APP: antimicrobial proteins and peptides
CRP: C-reactive protein
GBS: Group B streptococcus
LOC: lab-on-a-chip
PCR: polymerase chain reaction
TLR: Toll-like receptor
WHO: World Health Organization

## References

[1] Thaver D, Zaidi AK (2009). Burden of neonatal infections in developing countries: a review of evidence from community-based studies.. Pediatr Infect Dis J.

[2] Lawn JE, Cousens S, Zupan J (2005). 4 million neonatal deaths: when? Where? Why?. Lancet.

[3] Darmstadt GL, Bhutta ZA, Cousens S, Adam T, Walker N (2005). Evidence-based, cost-effective interventions: how many newborn babies can we save?. Lancet.

[4] Bahl R, Martines J, Ali N, Bhan MK, Carlo W (2009). Research priorities to reduce global mortality from newborn infections by 2015.. Pediatr Infect Dis J.

[5] Schuchat A, Zywicki SS, Dinsmoor MJ, Mercer B, Romaguera J (2000). Risk factors and opportunities for prevention of early-onset neonatal sepsis: a multicenter case-control study.. Pediatrics.

[6] Zaidi AK, Thaver D, Ali SA, Khan TA (2009). Pathogens associated with sepsis in newborns and young infants in developing countries.. Pediatr Infect Dis J.

[7] Stoll BJ (1997). The global impact of neonatal infection.. Clin Perinatol.

[8] Zaidi AK, Huskins WC, Thaver D, Bhutta ZA, Abbas Z (2005). Hospital-acquired neonatal infections in developing countries.. Lancet.

[9] Stoll BJ, Hansen N, Fanaroff AA, Wright LL, Carlo WA (2002). Changes in pathogens causing early-onset sepsis in very-low-birth-weight infants.. N Engl J Med.

[10] Seale AC, Mwaniki M, Newton CR, Berkley JA (2009). Maternal and early onset neonatal bacterial sepsis: burden and strategies for prevention in sub-Saharan Africa.. Lancet Infect Dis.

[11] Tiskumara R, Fakharee SH, Liu CQ, Nuntnarumit P, Lui KM (2009). Neonatal infections in Asia.. Arch Dis Child Fetal Neonatal Ed.

[12] Schrag SJ, Hadler JL, Arnold KE, Martell-Cleary P, Reingold A (2006). Risk factors for invasive, early-onset Escherichia coli infections in the era of widespread intrapartum antibiotic use.. Pediatrics.

[13] Menezes EV, Yakoob MY, Soomro T, Haws RA, Darmstadt GL (2009). Reducing stillbirths: prevention and management of medical disorders and infections during pregnancy.. BMC Pregnancy Childbirth.

[14] Stiehm ER, Fudenberg HH (1966). Serum levels of immune globulins in health and disease: a survey.. Pediatrics.

[15] Levy O (2007). Innate immunity of the newborn: basic mechanisms and clinical correlates.. Nat Rev Immunol.

[16] Levy O, Martin S, Eichenwald E, Ganz T, Valore E (1999). Impaired innate immunity in the newborn: newborn neutrophils are deficient in bactericidal/permeability-increasing protein.. Pediatrics.

[17] Levy O (2005). Innate immunity of the human newborn: distinct cytokine responses to LPS and other Toll-like receptor agonists.. J Endotoxin Res.

[18] Angelone DF, Wessels MR, Coughlin M, Suter EE, Valentini P (2006). Innate immunity of the human newborn is polarized toward a high ratio of IL-6/TNF-alpha production in vitro and in vivo.. Pediatr Res.

[19] Marodi L (2006). Neonatal innate immunity to infectious agents.. Infect Immun.

[20] Kramer MS (2000). Balanced protein/energy supplementation in pregnancy.. Cochrane Database Syst Rev.

[21] Haider BA, Bhutta ZA (2006). Multiple-micronutrient supplementation for women during pregnancy.. Cochrane Database Syst Rev.

[22] Healy CM, Baker CJ (2007). Maternal immunization.. Pediatr Infect Dis J.

[23] Demicheli V, Barale A, Rivetti A (2005). Vaccines for women to prevent neonatal tetanus.. Cochrane Database Syst Rev.

[24] Zaman K, Roy E, Arifeen SE, Rahman M, Raqib R (2008). Effectiveness of maternal influenza immunization in mothers and infants.. N Engl J Med.

[25] Sinha A, Lieu TA, Paoletti LC, Weinstein MC, Platt R (2005). The projected health benefits of maternal group B streptococcal vaccination in the era of chemoprophylaxis.. Vaccine.

[26] Quiambao BP, Nohynek H, Kayhty H, Ollgren J, Gozum L (2003). Maternal immunization with pneumococcal polysaccharide vaccine in the Philippines.. Vaccine.

[27] Bhutta ZA, Darmstadt GL, Hasan BS, Haws RA (2005). Community-based interventions for improving perinatal and neonatal health outcomes in developing countries: a review of the evidence.. Pediatrics.

[28] Rhee V, Mullany LC, Khatry SK, Katz J, LeClerq SC (2008). Maternal and birth attendant hand washing and neonatal mortality in southern Nepal.. Arch Pediatr Adolesc Med.

[29] Meegan ME, Conroy RM, Lengeny SO, Renhault K, Nyangole J (2001). Effect on neonatal tetanus mortality after a culturally-based health promotion programme.. Lancet.

[30] Biran A, Schmidt WP, Wright R, Jones T, Seshadri M (2009). The effect of a soap promotion and hygiene education campaign on handwashing behaviour in rural India: a cluster randomised trial.. Trop Med Int Health.

[31] Luby SP, Agboatwalla M, Painter J, Altaf A, Billhimer WL (2004). Effect of intensive handwashing promotion on childhood diarrhea in high-risk communities in Pakistan: a randomized controlled trial.. JAMA.

[32] Curtis VA, Danquah LO, Aunger RV (2009). Planned, motivated and habitual hygiene behaviour: an eleven country review.. Health Educ Res.

[33] McClure EM, Goldenberg RL, Brandes N, Darmstadt GL, Wright LL (2007). The use of chlorhexidine to reduce maternal and neonatal mortality and morbidity in low-resource settings.. Int J Gynaecol Obstet.

[34] Lumbiganon P, Thinkhamrop J, Thinkhamrop B, Tolosa JE (2004). Vaginal chlorhexidine during labour for preventing maternal and neonatal infections (excluding Group B Streptococcal and HIV).. Cochrane Database Syst Rev.

[35] Ohlsson A, Shah VS (2009). Intrapartum antibiotics for known maternal Group B streptococcal colonization.. Cochrane Database Syst Rev.

[36] (2005). Early-onset and late-onset neonatal group B streptococcal disease–United States, 1996-2004.. MMWR Morb Mortal Wkly Rep.

[37] www.aap.org accessed 3rd September 2009

[38] Mullany LC, Darmstadt GL, Khatry SK, Katz J, LeClerq SC (2006). Topical applications of chlorhexidine to the umbilical cord for prevention of omphalitis and neonatal mortality in southern Nepal: a community-based, cluster-randomised trial.. Lancet.

[39] Mullany LC, Darmstadt GL, Tielsch JM (2006). Safety and impact of chlorhexidine antisepsis interventions for improving neonatal health in developing countries.. Pediatr Infect Dis J.

[40] Darmstadt GL, Saha SK, Ahmed AS, Chowdhury MA, Law PA (2005). Effect of topical treatment with skin barrier-enhancing emollients on nosocomial infections in preterm infants in Bangladesh: a randomised controlled trial.. Lancet.

[41] Siegrist CA (2003). Mechanisms by which maternal antibodies influence infant vaccine responses: review of hypotheses and definition of main determinants.. Vaccine.

[42] Bhandari N, Bahl R, Mazumdar S, Martines J, Black RE (2003). Effect of community-based promotion of exclusive breastfeeding on diarrhoeal illness and growth: a cluster randomised controlled trial.. Lancet.

[43] Edmond KM, Zandoh C, Quigley MA, Amenga-Etego S, Owusu-Agyei S (2006). Delayed breastfeeding initiation increases risk of neonatal mortality.. Pediatrics.

[44] Edmond KM, Kirkwood BR, Amenga-Etego S, Owusu-Agyei S, Hurt LS (2007). Effect of early infant feeding practices on infection-specific neonatal mortality: an investigation of the causal links with observational data from rural Ghana.. Am J Clin Nutr.

[45] Mullany LC, Katz J, Li YM, Khatry SK, LeClerq SC (2008). Breast-feeding patterns, time to initiation, and mortality risk among newborns in southern Nepal.. J Nutr.

[46] Darlow BA, Graham PJ (2007). Vitamin A supplementation to prevent mortality and short and long-term morbidity in very low birthweight infants.. Cochrane Database Syst Rev.

[47] Gogia S, Sachdev HS (2009). Neonatal vitamin A supplementation for prevention of mortality and morbidity in infancy: systematic review of randomised controlled trials.. BMJ.

[48] English M, Ngama M, Mwalekwa L, Peshu N (2004). Signs of illness in Kenyan infants aged less than 60 days.. Bull World Health Organ.

[49] (2008). Clinical signs that predict severe illness in children under age 2 months: a multicentre study.. Lancet.

[50] Cromwell D, Joffe T, Hughes R, Murphy D, Dhillon C (2008). The local adaptation of national recommendations for preventing early-onset neonatal Group B Streptococcal disease in UK maternity units.. J Health Serv Res Policy.

[51] Buhimschi CS, Bhandari V, Hamar BD, Bahtiyar MO, Zhao G (2007). Proteomic profiling of the amniotic fluid to detect inflammation, infection, and neonatal sepsis.. PLoS Med.

[52] Buhimschi CS, Dulay AT, Abdel-Razeq S, Zhao G, Lee S (2009). Fetal inflammatory response in women with proteomic biomarkers characteristic of intra-amniotic inflammation and preterm birth.. BJOG.

[53] Neal PR, Kleiman MB, Reynolds JK, Allen SD, Lemons JA (1986). Volume of blood submitted for culture from neonates.. J Clin Microbiol.

[54] Benjamin DK, Miller W, Garges H, Benjamin DK, McKinney RE (2001). Bacteremia, central catheters, and neonates: when to pull the line.. Pediatrics.

[55] Kurlat I, Stoll BJ, McGowan JE (1989). Time to positivity for detection of bacteremia in neonates.. J Clin Microbiol.

[56] Darmstadt GL, Batra M, Zaidi AK (2009). Parenteral antibiotics for the treatment of serious neonatal bacterial infections in developing country settings.. Pediatr Infect Dis J.

[57] Peters RP, van Agtmael MA, Danner SA, Savelkoul PH, Vandenbroucke-Grauls CM (2004). New developments in the diagnosis of bloodstream infections.. Lancet Infect Dis.

[58] Moisi JC, Saha SK, Falade AG, Njanpop-Lafourcade BM, Oundo J (2009). Enhanced diagnosis of pneumococcal meningitis with use of the Binax NOW immunochromatographic test of Streptococcus pneumoniae antigen: a multisite study.. Clin Infect Dis.

[59] Jordan JA, Durso MB (2000). Comparison of 16S rRNA gene PCR and BACTEC 9240 for detection of neonatal bacteremia.. J Clin Microbiol.

[60] Jordan JA, Durso MB (2005). Real-time polymerase chain reaction for detecting bacterial DNA directly from blood of neonates being evaluated for sepsis.. J Mol Diagn.

[61] Jordan JA, Durso MB, Butchko AR, Jones JG, Brozanski BS (2006). Evaluating the near-term infant for early onset sepsis: progress and challenges to consider with 16S rDNA polymerase chain reaction testing.. J Mol Diagn.

[62] Weile J, Knabbe C (2009). Current applications and future trends of molecular diagnostics in clinical bacteriology.. Anal Bioanal Chem.

[63] Andrade SS, Bispo PJ, Gales AC (2008). Advances in the microbiological diagnosis of sepsis.. Shock.

[64] Jordan JA, Jones-Laughner J, Durso MB (2009). Utility of pyrosequencing in identifying bacteria directly from positive blood culture bottles.. J Clin Microbiol.

[65] Verboon-Maciolek MA, Thijsen SF, Hemels MA, Menses M, van Loon AM (2006). Inflammatory mediators for the diagnosis and treatment of sepsis in early infancy.. Pediatr Res.

[66] Franz AR, Steinbach G, Kron M, Pohlandt F (1999). Reduction of unnecessary antibiotic therapy in newborn infants using interleukin-8 and C-reactive protein as markers of bacterial infections.. Pediatrics.

[67] Edgar JD, Wilson DC, McMillan SA, Crockard AD, Halliday MI (1994). Predictive value of soluble immunological mediators in neonatal infection.. Clin Sci (Lond).

[68] Yager P, Edwards T, Fu E, Helton K, Nelson K (2006). Microfluidic diagnostic technologies for global public health.. Nature.

[69] Zhang Y, Ozdemir P (2009). Microfluidic DNA amplification–a review.. Anal Chim Acta.

[70] Stevens DY, Petri CR, Osborn JL, Spicar-Mihalic P, McKenzie KG (2008). Enabling a microfluidic immunoassay for the developing world by integration of on-card dry reagent storage.. Lab Chip.

[71] Thaver D, Ali SA, Zaidi AK (2009). Antimicrobial resistance among neonatal pathogens in developing countries.. Pediatr Infect Dis J.

[72] Darmstadt GL, Batra M, Zaidi AK (2009). Oral antibiotics in the management of serious neonatal bacterial infections in developing country communities.. Pediatr Infect Dis J.

[73] Bhutta ZA, Zaidi AK, Thaver D, Humayun Q, Ali S (2009). Management of newborn infections in primary care settings: a review of the evidence and implications for policy?. Pediatr Infect Dis J.

[74] Bang AT, Bang RA, Baitule SB, Reddy MH, Deshmukh MD (1999). Effect of home-based neonatal care and management of sepsis on neonatal mortality: field trial in rural India.. Lancet.

